# Association of rs780094 polymorphism of glucokinase regulatory protein with non-alcoholic fatty liver disease

**DOI:** 10.1186/s13104-020-4891-y

**Published:** 2020-01-10

**Authors:** Saba Mohammadi, Safar Farajnia, Masoud Shadmand, Fatemeh Mohseni, Roghayyeh Baghban

**Affiliations:** 1 Drug Applied Research Center, Tabriz University of Medical Sciences, Tabriz, Iran; 2 Biotechnology Research Center, Tabriz University of Medical Sciences, Tabriz, Iran; 3 Nutrition Research Center, Department of Community Nutrition, School of Nutrition, Tabriz University of Medical Sciences, Tabriz, Iran; 4 Immunology Research Center, Tabriz University of Medical Sciences, Tabriz, Iran

**Keywords:** NAFLD, rs780094 polymorphism, Biochemical parameters, Demographic parameters

## Abstract

**Objective:**

GCK rs780094 polymorphism is a single nucleotide polymorphism that has been associated with obesity, type II diabetes and dyslipidemia in some populations, conditions that highly related to NAFL etiology. The present study aimed to evaluate the relationship between NAFLD and rs780094 polymorphism in patients with NAFLD in Tabriz city, northwest of Iran. The rs780094 polymorphism was determined in 74 patients with NAFLD by PCR–RFLP technique. Demographic information was collected using a questionnaire and biochemical analysis was performed using standard laboratory methods.

**Results:**

There was a significant difference between case and control subjects for alanine aminotransferase, aspartate aminotransferase, HDL-C and triglycerides (*P *< 0.05). Analysis by PCR–RFLP method revealed that there were no significant differences between NAFLD and healthy subjects for rs780094 polymorphism in the study population. The results of this study indicated that rs780094 polymorphism is not associated with NAFLD in subjects from Tabriz city.

## Introduction

Non-alcoholic fatty liver disease (NAFLD), was first identified in 1980 by Ludwig et al., through fatty infiltration of the liver among non-alcoholic patients [1]. NAFLD is considered as a major public health concern due to increasing prevalence of obesity in both children and adults. The disease is diagnosed by hepatic steatosis or presence of fat in liver parenchyma without inflammation and also in the absence of excessive alcohol consumption. NAFLD is also known as a hepatic component of metabolic syndrome [2] which includes a broad range of clinic pathological conditions ranging from simple steatosis to non-alcoholic steatohepatitis (NASH) [3]. The progressed NASH is identified through cellular necrosis and inflammatory infiltration that can subsequently create complications such as cirrhosis, liver failure and hepatocellular carcinoma [4].

Genetic variants, as likely etiological factors, are investigated for NAFLD due to its familial clustering [5–8]. Some research studies have shown a strong correlation between usual missense variant (rs738409) in the patatin-like phospholipase domain-containing protein 3 (PNPLA3) gene and susceptibility to NAFLD and NASH [9].

Glucose-phosphorylating enzyme glucokinase is responsible for hepatic glucose metabolism and activation of hepatic lipogenesis. The expression of Glucokinase is regulated by GCKR [10]. It has shown that GCKR, encoded by GCKR gene, allosterically binds to GCK and regulates its activity. Genome-wide association studies (GWAS) have recognized a single-nucleotide polymorphisms (SNPs) which increases the risk of NAFLD. It has shown that rs780094 polymorphism of GCKR is related to triglyceride levels [8, 11, 12]. Also, an association between rs780094 polymorphism of GCKR and diabetes, particularly in Asian population has reported in a previous study [13]. Moreover, population studies showed that NAFLD is severely related to obesity, insulin resistance/type II diabetes mellitus and dyslipidemia [14–17]. Thus, we hypothesized the presence of a possible relationship between NAFLD and rs780094 polymorphism. The presence of a strong association between NAFLD, with diabetes mellitus and hepatic triglyceride accumulation and also an association between rs780094 polymorphism with diabetes and triglyceride levels supported our hypothesis. The present study investigated the relationship between NAFLD and rs780094 polymorphism in patients with NAFLD in subjects from Tabriz city in the north-west of Iran.

## Main text

### Materials and methods

#### Subjects and samples

This case–control study was conducted on 74 patients with NAFLD that were confirmed by both sonography and high levels of liver enzymes. Also, 72 subjects without NAFLD were considered as control. The patients were randomly selected from patients in Gastrointestinal clinics. The subjects were informed about the purpose of the study and written informed consent was obtained. The inclusion criteria were similar in the case and control subjects, as follows; mean age 20–50 years and mean body mass index (BMI) 25–39.9 kg/m^2^. The exclusion criteria were also as follows; age ranging > 50 years and < 20 years, BMI < 25 kg/m^2^, having hemochromatosis, Wilson and autoimmune diseases, tobacco and alcohol consumption, the use of hepatotoxic and psychotic drugs, weight loss diets over last 3 months, gland disorders, pregnancy and lactation, intestinal bypass surgery, acute and chronic liver diseases and viral hepatitis.

#### Samples preparation and biochemical analysis

Blood samples were collected from case and control subjects and transferred to molecular biology laboratory of biotechnology research center. The biochemical parameters consisting of alanine aminotransferase (ALT), aspartate aminotransferase (AST), low density lipoprotein (LDL) cholesterol, high density lipoprotein (HDL) cholesterol, total cholesterol, and plasma triglyceride level was analyzed using standard laboratory techniques.

#### Collection of anthropometric data

The information related to age, sex, drug consumption, cigarette smoking, etc. were collected by questionnaire. BMI was defined as weight/height^2^ (kg/m^2^). Waist and hip circumference and waist-to-hip ratio were also measured.

#### DNA extraction and genotyping

DNA was extracted from blood samples using salting-out method. Briefly, 500 µl of blood samples were transferred to the 15 ml tubes and the red blood cells were lysed by washing three times with lysis buffer (155 mm NH_4_Cl, 10 mM KHCO_3_, 10 mM EDTA). Then, 500 µl of DNG-Plus reagents (Cinnagen co, Iran) was added to the cell pellet and slowly stirred to obtain the homogenized sample. DNA was then precipitated by 350 µl of isopropanol, washed with ethanol 75% and dissolved in 50 µl distilled water. The extracted DNA was analyzed using electrophoresis on 1% agarose gel and spectrophotometry.

RFLP-PCR was used to determinate the genotypes of subjects for rs738409 polymorphism. The DNA extracted from subjects was amplified with the following primers, Forward: 5′-AGCTCACGCTGGAACTTCTG-3′ and Reverse: 5′-CATGTTGGCTAGGCTTGTTGAG-3′, using Taq DNA polymerase. PCR was performed in a final volume of 25 µl including; 10 pm of each primer, 100 ng of genomic DNA, 1 U of Taq DNA polymerase and 22 µl of PCR master mix. The PCR program for DNA amplification was as follows; initial denaturation at 94 °C for 4 min, 35 cycles of denaturation at 94 °C for 1 min, annealing at 53 °C for 1 min, extension at 72 °C for 45 s and final extension at 72 °C for 5 min. After PCR, 10 µl of PCR products were digested with 1 U of PscI enzyme for 1 h at 37 °C and visualized on 2% agarose gel, after staining with safestain. The 100-bp DNA ladder was used for determining the size of digested product. After electrophoresis, genotypes GG at 311 bp, CC at 194 bp and 117 bp and CG at 311 and 194 bp were detected.

#### Statistical analysis

Statistical analysis was performed using SPSS software (Statistical Package for the Social Sciences) Version 23. Qualitative and quantitative data were expressed as Mean (± standard deviation) and frequency (%), respectively. The Mann–Whitney U test and independent t-test were used for analysis of the data. *P *< 0.05 was considered significant.

### Results

#### Demographic and clinical characteristics

Demographic and clinical data of the subjects have been demonstrated in Table 1. There were significant differences between control and case group for HDL-C, triglycerides, ALT and AST (*P *< 0.05), so that patients with NAFLD showed higher concentrations of triglycerides, ALT and AST and lower concentration of HDL-C compared to control group (*P *< 0.05).

**Table 1:** Demographic and clinical data of the subjects

Characteristics	Control (N = 72)	Case (N = 74)	*P*
Sex (men %)	36.1	48.6	ns
Sex (women %)	63.9	51.4	ns
Age (years)	38.88 ± 8.25	40.54 ± 8.42	ns
BMI (kg/m^2^)	31.51 ± 4.09	31.90 ± 4.18	ns
Waist circumference (cm)	100.2 ± 8.75	103.36 ± 9.31	ns
Hip circumference (cm)	111.86 ± 8.05	112.16 ± 8.13	ns
Waist-to-hip ratio	0.897 ± 0.065	0.922 ± 0.052	ns
Weight (kg)	84.66 ± 12.15	86.44 ± 11.14	ns
Height (cm)	164.04 ± 10.19	164.96 ± 10.49	ns
Cholesterol (mg/dl)	187.40 ± 29.33	183.08 ± 37.03	ns
Triglycerides (mg/dl)	140.71 ± 75.84	173.55 ± 76.33	*
HDL-C (mg/dl)	48.08 ± 11.78	43.09 ± 11.43	*
LDL-C (mg/dl)	110.98 ± 26.64	105.03 ± 34.75	ns
ALT (IU/l)	26.29 ± 9.41	49.99 ± 26.16	*
AST (IU/l)	22.97 ± 6.11	32.99 ± 14.97	*
Fasting glucose (mg/dl)	90.17 ± 10.16	90.47 ± 11.27	ns

*P < 0.05; *ns* non-significant or *P* > 0.05

#### Genotypic and allelic frequency of rs780094 polymorphism of GCKR

The distribution of the GCKR rs780094 genotype for the case and control groups has shown in Table 2. Analysis of the results indicated that there was no significant difference between case and control groups for CT and TT genotypes. However, compared to the normal healthy control subjects, the frequency of CC genotypes was significantly lower in NAFLD patients (Table 2).

**Table 2:** Genotypic and allelic frequency (%) of GCKR rs780094

	Control (N = 72)	Case (N = 74)	*P*
CC genotype	31.9	18.9	ns
CT genotype	47.2	48.6	ns
TT genotype	20.8	32.4	ns
T allele	0.44	0.57	ns
C allele	0.56	0.43	ns

*P < 0.05; *ns* non-significant or *P* > 0.05

#### Relation of demographic and clinical characteristics with GCKR rs780094

Table 3 shows a correlation between demographic and clinical characteristics of subjects and GCKR rs780094 genotype. Logistic regression showed that there was not any correlation between demographic and clinical characteristics and GCKR rs780094 genotype; indicating lack of significant role of GCKR rs780094 genotype in NAFLD in the study population.

**Table 3:** Relation of demographic and clinical characteristics with GCKR rs780094 genotype in patients with NAFLD (N = 74)

Characteristics	CC	CT	TT	β coefficient	*P*
Sex (men %)	64.3	38.9	50	–	ns
Sex (women %)	35.7	61.1	50	–	ns
Age (years)	40.43 ± 8.67	42.58 ± 8.10	38.29 ± 8.53	− 0.027	ns
BMI (kg/m^2^)	30.70 ± 3.05	31.30 ± 4.19	32.16 ± 4.59	0.057	ns
Waist circumference (cm)	100.0 ± 7.33	106.0 ± 10.91	101.0 ± 7.53	− 0.033	ns
Hip circumference (cm)	108.0 ± 7.14	115.0 ± 8.36	111.0 ± 7.10	0.011	ns
Weight (kg)	82.44 ± 9.56	88.78 ± 11.59	88.78 ± 11.59	0.136	ns
Height (cm)	167.43 ± 8.93	163.28 ± 10.81	165.0 ± 10.50	0.103	ns
Cholesterol (mg/dl)	176.64 ± 45.27	185.14 ± 35.06	182.17 ± 33.75	− 0.037	ns
Triglycerides (mg/dl)	184.71 ± 68.55	172.64 ± 88.37	187.08 ± 39.37	0.007	ns
HDL-C (mg/dl)	43.00 ± 10.94	43.97 ± 11.79	43.08 ± 11.07	− 0.007	ns
LDL-C (mg/dl)	96.70 ± 40.81	107.77 ± 29.91	101.67 ± 35.29	0.051	ns
ALT (IU/l)	52.21 ± 20.25	44.11 ± 29.10	56.75 ± 23.80	0.012	ns
AST (IU/l)	36.29 ± 10.97	29.61 ± 12.41	36.67 ± 19.04	− 0.010	ns
Fasting glucose (mg/dl)	90.57 ± 8.57	92.06 ± 10.08	88.00 ± 13.32	− 0.005	ns

*ns* non-significant or *P* > 0.05

### Discussion

Genome-wide association studies have shown that polymorphisms in the glucokinase regulatory protein are related to triglyceride level, obesity, insulin resistance/type II diabetes mellitus and dyslipidemia [14–17], common conditions associated with NAFLD disorder. Therefore, we aimed to investigate the relationship between the GCKR rs780094 variant and NAFLD in patients in the city of Tabriz, northwest of Iran.

The results of the current study indicated that TT genotype had a higher frequency in NAFLD cases compared to the control group, although this difference was not significant (*P *> 0.05). This finding was against a report from china. Yang et al. in 2011. [11] reported that the GCKR variants could be considered as a risk factor for NAFLD; however their control study group was not obese.

A meta-analysis has also shown that GCKR rs780094 T allele is a protective factor against diabetes and obesity [11]. Some other studies have shown a significant association between GCKR rs780094 and NAFLD [18]. However, the lack of relationship between the GCKR rs780094 genotype and the NAFLD, in the present study, might be attributed to the different ethnic background of the studied groups. In the current study, there was no association between GCKR rs780094 and anthropometric characteristics of the patients. It has known that anthropometric parameters not only related to the genetic background but also dependent on lifestyle. In a study on Iranian population, it has been shown a relationship among NAFLD, age, gender, obesity, metabolic syndrome, triglycerides, higher glucose, blood pressure and waist circumference [19]. Bhatt et al. [20] reported lack of any significant correlation between age and NAFLD in Asian Indians. Consistent with our findings, Lin et al. [21] in a study on Taiwanese children reported lack of significant association between NAFLD, age, BMI and waist circumference.

ALT and AST are known as liver biomarkers and liver injuries in NAFLD can disturb ALT and AST levels. Moreover, liver is known as a metabolizing organ for lipids, thus NAFLD, as a liver disorder, can disturb lipid levels. Our results indicated significant changes in HDL-C, triglycerides, AST and ALT as risk factors. In agreement with our results, Bhatt et al. [20] and Hotta et al. [22] reported that ALT and AST were significantly higher in NAFLD group compared to healthy controls in Asian populations.

In contrast to our findings, Petta et al. [23] reported that HDL, triglycerides, cholesterol and ALT did not show significant difference between case and control groups. In another investigation, Shen et al. [24] could not find significant difference for fasting glucose, cholesterol and triglycerides.

These discrepancies in findings of different studies might be attributed to the variations in the stage of disease, sample size and different ethnic background.

### Conclusion

The results of our study indicated that GCKR rs780094 TT genotype is not responsible for NAFLD in the studied population. Our results also showed that age, sex, BMI, cholesterol, fasting glucose, LDL-C, waist circumference, hip circumference and waist-to-hip ratio were not significantly different in case and control groups. Changes in triglycerides, ALT, AST, and HDL-C were found as risk factors of NAFLD in the study groups. Further studies with larger sample size could be helpful in holding more relevant findings and interventional designs are needed to determine the effects of dietary compounds in different GCKR genotypes in nonalcoholic fatty liver disease.

### Limitations

In the present manuscript, we explored the association of rs780094 polymorphism in GCKR with NAFLD and couldn’t find any association. Therefore, these results are specifically valid for the study population and its generalization to other population needs further studies.

## Data Availability

All data generated or analysed during this study are included in this published article.

## Abbreviations

PCR: polymerase chain reaction
RFLP: fragment length polymorphism
NAFLD: non-alcoholic fatty liver disease
PNPLA3: phospholipase domain-containing protein 3
NASH: non-alcoholic steatohepatitis
GCKR: glucokinase regulatory
SNPs: single-nucleotide polymorphisms
GWAS: genome-wide association studies
ALT: alanine aminotransferase
AST: aspartate aminotransferase
LDL: low density lipoprotein
HDL: high density lipoprotein
